# Supplementary material for: Content-rich biological network constructed by mining PubMed abstracts
Source: BMC Bioinformatics. 2004 Oct 8;5:147. doi: 10.1186/1471-2105-5-147 (PMC528731; doi:10.1186/1471-2105-5-147)
Supplement: Additional File 5 — The original Chilibot query results of the term "long-term potentiation (LTP)" and 22 other terms, limiting the latest references analyzed to the years 1990, 1995, 2000, and 2004. [file 1471-2105-5-147-S5.bz2 › chilibotAdditionalFile5/ltp1995/html/AMPA_SYNAPTOPHYSIN.html]

 


 **AMPA** and **SYNAPTOPHYSIN** 
  
Found 4 abstracts in PubMed,  **4 abstracts were retrieved and analyzed**.  


---

 Search Google  |
 PDF files only 
|  EDU domain only 

---

**Interactive relationship** (e.g. stimulation, inhibition, etc)

**Parallel relationship** (e.g. studied together, co-existance, homology, etc.)

- Long term maintenance was evident for other synapse related proteins, including  **synaptophysin** , neural cell adhesion molecule isoforms, and an  **AMPA**  receptor related antigen, as well as for certain structural and cytoskeletal components.  Ref: 8773255 Hippocampus, 1995
- Since the nerve terminal markers  **synaptophysin**  and the SV2 glycoprotein were not detectably different in the two groups of mice, the age related reduction in  **AMPA**  receptors is not likely to be due to a general decrease in synaptic density.  Ref: 1327416 Brain Res, 1992
